# Beyond Loss of Kidney Function: Patient Care in Autosomal Dominant Polycystic Kidney Disease

**DOI:** 10.34067/KID.0000000000000296

**Published:** 2023-11-27

**Authors:** Marie C. Hogan, Kathryn Simmons, Lawrence Ullman, Maryam Gondal, Neera K. Dahl

**Affiliations:** 1 Division of Nephrology and Hypertension, Mayo Clinic, Rochester, Minnesota; 2 Section of Nephrology, Yale University School of Medicine, New Haven, Connecticut

**Keywords:** ADPKD, liver cysts

## Abstract

Patients with autosomal dominant polycystic kidney disease benefit from specialized care over their lifetimes, starting with diagnosis of the condition with ongoing discussion of both the renal course and extra-renal issues. Both renal and extra-renal issues may continue to cause major morbidity even after successful kidney transplant or initiation of RRT, and extra-renal disease aspects should always be considered as part of routine management. In this review, we will focus on updates in pain/depression screening, cardiac manifestations, liver and pancreatic cysts, kidney stone management, and genetic counseling. In some instances, we have shared our current clinical practice rather than an evidence-based guideline. We anticipate more standardization of care after the release of the Kidney Disease Improving Global Outcomes guidelines for management in autosomal dominant polycystic kidney disease later this year.

## Introduction

Autosomal dominant polycystic kidney disease (ADPKD) patient care is often complex and multidisciplinary^1^ because ADPKD is a systemic disease affecting multiple organ systems.^2^ This review is not intended to be a comprehensive guide to management of all extra-renal ADPKD manifestations. Instead, we have chosen to highlight those areas in which there are advances affecting management of patients. We will cover pain and depression screening, cardiac manifestations of ADPKD, screening for intracranial aneurysms (ICAs) and vascular aneurysms, and management of liver and pancreatic cysts. Because genetic testing in cystic kidney disease is rapidly being adopted in practice, we offer some general suggestions on ordering and interpreting testing. We anticipate more standardization of care after the release of the Kidney Disease Improving Global Outcomes guidelines for management in ADPKD later this year and in the United States with harmonization of practice in the polycystic kidney disease (PKD) Foundation designated Centers of Excellence.

## Pain and Depression

ADPKD significantly affects health-related quality of life.^3,4^ Pain is a primary driver of the mental and physical burden of the disease, contributing substantially to reductions in health-related quality of life.^5–7^ Pain related to increasing kidney size and cyst complications is reported in 50% of patients with ADPKD, with 20% reporting pain often, usually, or always.^6^ Pain remains an issue as disease progresses with 44% of transplant and 59% of dialysis patients reporting pain.^3^ Pain associated with ADPKD carries a physical and mental burden and is described by patients as inexplicable and unpredictable in qualitative studies,^5^ serving as a reminder of patients' diseased state.^7^ Patients desire support from their providers, open and honest conversations regarding pain management, and information and resources to learn more about their condition.^8^

Despite patients reporting pain management as a top priority in ADPKD outcomes,^9^ many feel that their pain is underestimated by their physician and wish for more discussion surrounding pain management and strategies.^3,7^ The ADPKD Pain and Discomfort Scale was developed and validated to specifically evaluate the severity and type of pain experienced by patients with ADPKD.^10^ Although this tool was designed for standardization of patient-reported outcomes research, routine questioning could normalize discussion of pain^11^ and could provide nephrologists with the ability to monitor changes in pain severity and interference over time.

We propose asking about each pain type (sharp, dull, or fullness or discomfort) at each clinical encounter (Table 1), with further exploration of the domains of severity or interference^10^ with a positive response as a way of engaging in a discussion of individual patient concerns.

**Table 1:** Suggested screening at every encounter

PKD-Specific Concerns	Screening	Additional Comments
Pain^10^	Ask about change in chronic pain, acute pain, and fullness and discomfort	If positive, review the additional questions from ADPKD-PDS
Depression^12^	Ask the following two questions: During the past month have you often been bothered by feeling down, depressed, or hopeless? During the past month have you often been bothered by little interest or pleasure in doing things?	If positive, consider further screening, referral, and/or initiation of treatment
High BP^13^	We encourage patients to check BP at home, with a goal BP of 110/75 for younger high-risk patients and 120/80 for older patients with kidney function decline	We avoid use of diuretics for those patients considering or taking tolvaptan
Diet and lifestyle^14^	For patients with preserved kidney function, we encourage a low salt diet, high in potassium, with 3 L of fluid intake, and maintenance of an ideal body weight	For those patients interested in alternative strategies, we encourage intermittent fasting, with continued fluid intake but discuss concerns about high fat (ketogenic) diets

ADPKD, autosomal dominant polycystic kidney disease; ADPKD-PDS, ADPKD Pain and Discomfort Scale.

Between 22% and 60% of patients with ADPKD have depression,^15–17^ and therefore, we also recommend screening. Although providers generally consider early ADPKD to be asymptomatic, the mental impact of an ADPKD diagnosis is immediate. On diagnosis, patients deal with feelings of uncertainty, fear, and a sense of loss.^8^ Furthermore, patients report lower satisfaction with life compared with healthy individuals even at the early stages of disease.^18^ Patients express guilt surrounding passing the condition to their children, and for those without children, ADPKD can complicate the decision to have a child.^15^ Tolvaptan, approved for treatment of ADPKD in 2018, has been shown to decrease symptoms of depression^19^; however, other studies have shown that patients maintain the same kidney-related quality-of-life concerns as untreated patients, despite improved treatment satisfaction.^20,21^

Depression is also associated with worse physical health of patients with ADPKD. Patients with depression are more likely to deal with pain and have lower scores on quality-of-life questionnaires and less sleep.^15^ Patients with more symptoms are less likely to comply with dietary guidelines, which may negatively affect disease progression, and depression is more common in patients with more severe disease.^17^ Patients in the Chronic Renal Insufficiency Cohort were more likely to have rapid nonlinear eGFR decline with moderate or high scores on the Beck Depression Index compared with patients with low scores.^22^

Given the negative effects of depression on mental and physical health of patients with ADPKD, it is important to screen for depression early on in ADPKD disease course and at regular intervals throughout management. The US Preventative Services Taskforce recommends that all adults should be screened for depression using evidence-based protocols, such as various iterations of the Patient Health Questionnaire.^23^ A short two-question survey should be sufficient in first-line screening (Table 1).^12^ If positive, this should be followed-up with more comprehensive depression measures.

## Genetic Testing

Undoubtedly, genotype is an important predictor of clinical progression, but genetic testing is an evolving area in ADPKD. Opinion-based standards recommend genetic testing for patients with an unknown family history, with atypical imaging, for screening a young (younger than 40 years^24^) family member who wishes to be a kidney donor, and for use of in vitro fertilization with preimplantation genetic testing for mutation.^24,25^ Preimplantation genetic testing for mutation for ADPKD is possible if genetic studies in the affected parent are completed and conclusive before conception with cumulative success rates per couple of 58%–65%.^26,27^

The PKD spectrum of genes causing cystic diseases has expanded. Gene variants can be identified using specific targeted next-generation sequencing panels which are optimized for sequencing coding regions (exomes) of cystic kidney genes. The widespread availability of relatively inexpensive targeted next-generation sequencing panels has permitted rapid adoption into clinical practice. While this is welcome because many different genotypes may lead to a similar phenotype^28^ and knowing the genetic diagnosis may become a standard component of care, decisions regarding risk of progression (loss of kidney function) or screening for extra-renal features should not be made on the basis of genotype alone. Many nephrologists currently lack training in molecular genetic test interpretation. Geneticists, genetic counselors experienced in renal diseases, and nephrologists trained in clinical genomics will likely fill these gaps in the future.

## Liver Cysts

The liver is the most common extra-renal site of disease involvement in ADPKD. Most patients with polycystic liver disease (PLD) are asymptomatic and do not require treatment. Both liver cysts and parenchymal enlargement are responsible for most complications associated with PLD. The minority with symptomatic PLD complain of heartburn due to gastroesophageal reflux, early satiety, abdominal pain, abdominal distention, and so on^29^ (Table 2). Liver cyst infection can be life-threatening, and in suspected cases it is evaluated with blood cultures, possible cyst aspiration, and by positron emission tomography/computed tomography (CT) and treated with broad-spectrum, lipid-soluble antibiotics.^30^

**Table 2:** Potential liver complications in autosomal dominant polycystic kidney disease

Acute Complications from Single Cysts	Severe Hepatomegaly	Other Liver Complications
Infection or torsion Rupture or hemorrhage Obstructive jaundice Ascending cholangitis Biliary peritonitis (after cyst rupture)	Abdominal fullness Abdominal distension GERD/anorexia/nausea/early satiety Dyspnea Abdominal hernia LFT abnormalities Ascites, hepatic outflow obstruction, portal hypertension, IVC compression, bile duct compression Pleural effusion Organ displacement Failure to thrive/malnutrition Budd-Chiari syndrome	Isolated common bile duct dilation Hepatic fibrosis or biliary fibroadenomatosis Idiopathic biliary tract dilation (Caroli syndrome)

GERD, gastroesophageal reflux disease, LFT; liver function test; IVC, inferior vena cava.

Known risk factors influence PLD severity, including female sex, parity, and exogenous estrogen exposure, and cysts are more prevalent, and cyst burden is generally higher in women than in men. Women who have multiple pregnancies or who have used oral contraceptives or estrogen replacement therapy have more severe disease, suggesting an estrogen effect on hepatic cyst growth.^31–34^ Growth in liver cysts declines after menopause.^35,36^ A history of exposure to estrogen-containing oral contraceptives correlated with PLD growth in premenopausal women. Every year of exposure correlated with a 1.45% higher height-adjusted total liver volume (hTLV), which corresponded to a 15.5% higher hTLV for every 10 years of use compared with unexposed women^37^ although there was no association between estrogen-containing oral contraceptive use and hTLV in the combined group of both premenopausal and postmenopausal women or in the postmenopausal subgroup.

Although there is an association with estrogen and liver cysts, two recent analyses of younger patients, either from Consortium of Radiologic Imaging Study of PKD or HALT Progression of Polycystic Kidney Disease Cohort A, did not find an independent effect of pregnancy or exogenous estrogen use on liver volume.^36,38^ This discrepancy may be because liver cyst complications still affect only a minority of patients, and average liver volume was only slightly increased in most patients, or because other factors drive liver cyst growth. Liver cyst volume adjusted for height and age and then stratified maybe a predictive biomarker to identify individuals at highest risk for progressive liver cyst disease burden.^36^ We discuss the use of hormonal therapy with our female patients, with a recommendation for avoidance for those at higher risk of PLD progression and individualized counseling for those at lower risk. Conservatively, imaging consistent with a total liver volume <1500 ml, or only a few cysts seen by CT or magnetic resonance imaging (MRI) imaging, maybe considered lower risk. Antiestrogen therapies are currently in clinical trials^39^

Somatostatin analogs administered by intramuscular injection (octreotide, lanreotide) are effective in decreasing liver growth and may be considered for symptomatic patients, for whom surgical intervention is not appropriate.^40^ A meta-analysis of pooled data from 592 patients shows that these treatments are effective in decreasing total liver and kidney volume; however, side effects include higher blood sugars, diarrhea, abdominal pain, cholelithiasis/cholecystitis, and alopecia.^41^ Younger patients^42^ with an elevated alkaline phosphatase^43^ are more likely to respond, with discontinuation of therapy if there is no symptomatic benefit after 6 months. We have occasionally tried short-acting subcutaneous twice daily dosing of octreotide for patients who are not able to obtain the long-acting (monthly) preparations because of insurance issues.

**Figure 1:**
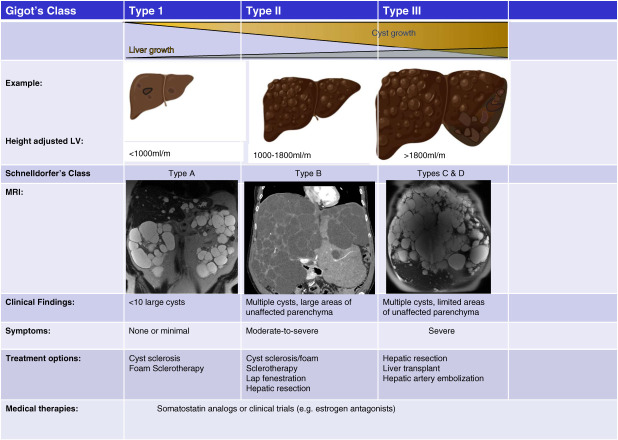
**Classification criteria for PLD severity, clinical presentation, symptoms, and treatment options.** Gigot and Schnelldorfer classifications are based on the number and size of the cysts and the remaining liver parenchyma. Height adjusted liver volume criteria: <1000, 1000–1800, >1800 ml/m. Coronal T2-weighted MRI images are shown for each patient type. In type A, the patient has large kidney cysts, but well-preserved liver parenchyma; in the type B patient, there is a residual preserved area of liver parenchyma; and in the type C and D patient, there is very little nonaffected liver. LV, left ventricle; MRI, magnetic resonance imaging; PLD, polycystic liver disease.

**Figure 2:**
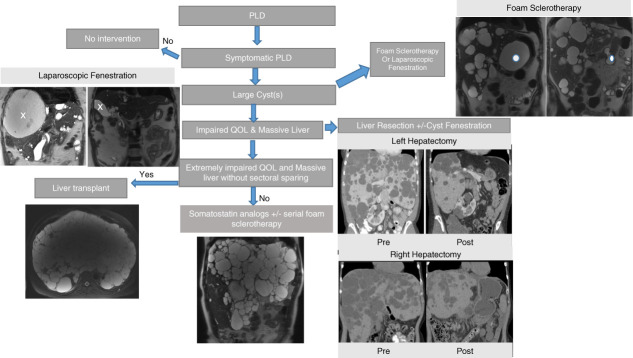
**Algorithm for PLD management in ADPKD.** Asymptomatic or mildly symptomatic patients may require no long-term intervention. Patients who develop impaired quality of life may benefit from foam sclerotherapy, laparoscopic fenestration, liver resection (partial hepatectomy), or liver transplant. Patients who are not candidates for surgical treatment may benefit from a trial of somatostatin analogs with or without serial foam sclerotherapy. Coronal T2-weighted MRI images are shown. The x and the dot show the changes in a cyst before and after therapy. ADPKD, autosomal dominant polycystic kidney disease.

Several imaging classification systems are used in PLD (Figure 1); Gigot classification and Mayo Clinic classification stratify patients for operative procedure selection.^44^ Cyst aspiration is indicated for large symptomatic liver cysts (Figure 2). Percutaneous aspiration may be followed by injection of a sclerosing agent which causes destruction of the epithelial lining inhibiting fluid production. Several centers have adopted foaming sclerosants^45,46^ providing superior long-term efficacy and the procedure being less painful than other sclerosants. Without sclerosant, the cyst fluid may re-accumulate.

Fenestration surgery permits surgical deroofing of multiple cysts in a single laparoscopic procedure but is associated with significant side effects (ascites, pleural effusion, bleeding, biliary leakage).^47^ Segmental liver resection (with or without cyst fenestration) could be considered in individuals with symptomatic disease and/or massive hepatomegaly, who harbor cyst rich segments and have at least one segment with unaffected liver parenchyma.^40,47^ Careful preoperative selection and imaging is needed to select appropriate patients (Figure 2).

Liver transplantation is warranted in patients with extremely debilitating symptoms, seriously compromised functional status and reduced quality of life, untreatable complications, such as portal hypertension, and severe malnutrition with bilobar extensive cystic liver disease lacking adequate areas of parenchymal sparing. Since patients with PLD have essentially normal liver function, calculated model for end-stage liver disease (MELD) scores are low or normal, and hence United Network for Organ Sharing has developed a MELD exemption review process for PLD cases. An important update in 2021 allowed for liver transplant listing for patients with a GFR <20 ml/min or with a prior kidney transplant or with moderate-to-severe malnutrition, in addition to those on dialysis.^48^ This change expanded eligibility, as previously only patients on dialysis were eligible for MELD exception points for liver transplant listing. In individuals with GFR <30 or ESKD, simultaneous deceased donor combined liver-kidney transplant is the most optimal approach. A recent single-center study shows good survival outcomes for orthotopic liver transplant alone, living donor liver transplant, and simultaneous liver-kidney transplant.^49^

## Pancreatic Lesions

Pancreatic cyst lesions are a recognized component of the ADPKD phenotype (seen in 15%–23% of patients)^50^ Most are incidental. As our practice has shifted to ordering more volumetric measurements of kidney and liver size, we have also frequently encountered incidental intraductal papillary mucinous neoplasms (IPMNs) in our patients with ADPKD (about 1% of patients). In general, these should be followed using standard protocols as developed for the general population. In a single-center review, IPMN patients were older, and seven of 12 had received kidney transplants.^50^ Thus, we suggest continued follow-up of patients with known IPMNs after kidney transplant, with appropriate surgical referral as indicated.

## Diverticulosis and Hernias

The prevalence of colonic diverticulosis increases with increasing age in both ADPKD and non-ADPKD populations. A recent large retrospective study found that prevalence of diverticulosis was higher in patients with an ADPKD diagnosis, compared with those without, with a median age at onset of 64 years, compared with 72 years in those without an ADPKD diagnosis.^51^ Diverticulitis was also more prevalent in those patients with an ADPKD diagnosis, with a higher prevalence of diverticulitis in patients with an ADPKD diagnosis and a kidney transplant, compared with patients with a kidney transplant and no ADPKD diagnosis.^51^ It is reasonable, therefore, to have a high degree of suspicion for diverticular complications particularly in older patients or patients with ESKD from ADPKD.

Abdominal wall hernias are more common in ADPKD compared with other kidney disease or general surgical patients.^52^ Patients with ADPKD with ESKD on CAPD are at increased risk for indirect inguinal hernia^53^; however, in our experience many patients with ADPKD do well on peritoneal dialysis.

## Cardiac Manifestations

Cardiac dysfunction is common in ADPKD because of both the activation of renin-angiotensin-aldosterone system and the presence of polycystins in vascular and cardiac tissue. A recent editorial reviewed many aspects of cardiac issues in ADPKD,^54^ and therefore, we have only highlighted a few areas for discussion below.

## Hypertension and Left Ventricular Hypertrophy

Hypertension in ADPKD is associated with an increased risk of renal loss^55^ with onset well before declining kidney function. The onset of hypertension before age 35 years is a clinical risk factor for progression in the Predicting Renal Outcome in PKD risk score.^56^ ADPKD adults are more likely to be nondippers without physiologic drop in BP during 24-hour ambulatory monitoring than age-matched normotensive healthy controls, and 52% of children with ADPKD were characterized as nondippers.^57,58^ In patients with preserved renal function, a morning BP surge, or a swift rise in BP in the early hours of the morning, was independently associated with left ventricular hypertrophy (LVH).^59^

In the era before the widespread use of angiotensin converting enzyme inhibitors or angiotensin receptor blockers, LVH in ADPKD was common^60^; however, the prevalence of LVH was low at baseline in the HALT Progression of Polycystic Kidney Disease cohort, perhaps reflecting the widespread use of these medications.^61^ Two recent studies^62,63^ have found higher rates of LVH, perhaps reflecting differences in LVH measurement. Obesity, insulin resistance, or polycystin-dependent pathways may affect cardiac outcomes.^64^ We do not routinely screen with echocardiograms for hypertension management in ADPKD.

We advocate BP measurement and counseling at every clinical encounter with a BP target of 110/75 for high-risk patients with preserved kidney function^13^ and 120/80^65^ for patients with renal decline (Table 1). We also encourage home BP monitoring (2–3 times a week) particularly during medication titration. Because salt intake is also associated with hypertension and kidney function decline in ADPKD,^66^ we review dietary guidelines as well. For patients age older than 40 years, without diagnosed atherosclerotic cardiovascular disease, we use the American College of Cardiology risk estimator tool to determine the need for additional preventative therapy, such as a statin or aspirin.^67^

## Valvular Abnormalities

Heart valve abnormalities are common in ADPKD affecting up to 39.5% of patients, with tricuspid valve regurgitation (16%), mitral valve regurgitation (15.3%), aortic valve regurgitation (4.8%), and mitral valve prolapse in 3.4%.^68^ Other studies have reported higher rates of mitral valve abnormalities ranging from 26% to 63% of patients.^63,69^ Aortic root aneurysms/dissection are more common in ADPKD,^70^ and patients with more rapidly progressive kidney disease (higher Mayo imaging classification) have larger aortic root diameters.^63^ Our practice is to offer a screening echocardiogram as part of initial screening only if there is a murmur on physical examination. If aortic root dilation is found, cardiac MRI or multidetector CT may be needed for further evaluation.

All our patients undergo echocardiograms as part of pretransplant evaluation regardless of physical examination findings as they approach kidney failure (Table 3).

**Table 3:** Suggested screening at initial encounter or at preparation for RRT

Screening	Initial Encounter	At Preparation for RRT
Cardiac	ECHO only if physical examination is abnormal	ECHO routinely
ICAs	MRA for positive family history, symptoms, or patient preference	Discuss MRA if not already performed
AAA		Men age 65–75 years with a smoking history or men older than 60 years with a family history^71^
Liver cyst burden	Consider measuring liver volume at the time that total kidney volume measurements are obtained. Risk assessment may help tailor discussion of hormonal therapy	Re-evaluate liver cyst burden to assess need for possible liver transplant in the future
Kidney stones	24-h urine with discussion of citrate therapy if hypocitraturia present	

ECHO, echocardiogram; ICA, intracranial aneurysm; MRA, magnetic resonance angiogram; AAA, abdominal aortic aneurysm.

## Pericardial Effusion/Cardiomyopathy

The incidence of pericardial effusion is increased in ADPKD.^72,73^ Up to 35% of patients with clinically diagnosed ADPKD and serum creatinine ≥1.1 mg/dl were found to have pericardial effusion, frequently moderate to large but without clinical consequence.^73^ Importantly, these effusions were not associated with degree of loss of renal function. Clinically, if no other cause (uremia or infection for instance) is found for a pericardial effusion, we monitor as clinically indicated.

Either *PKD1* or *PKD2* variants may be associated with cardiomyopathy,^74^ but idiopathic dilated cardiomyopathy is more commonly associated with PKD2 disease-causing variants.^74,75^ Although most medications for management of heart failure are safe in ADPKD, sodium-glucose cotransporter-2 inhibitor (SGLT2i) could be used after a careful consideration of the individual risks and benefits for a patient.^76^Treatment with an SGLT2i may stimulate vasopressin and thus be detrimental patients with for ADPKD. In patients with ADPKD with a low ejection fraction, or at high risk of death, SGLT2i may be beneficial. A pilot feasibility study (NCT05510115) will evaluate the safety of SGLT2i in ADPKD.

## Kidney Stone Management

Nephrolithiasis is common in ADPKD; the prevalence of stones is estimated to be 5–10 times greater in patients with ADPKD than in the general population.^77^ Patients with ADPKD are at a greater likelihood of having a hospital encounter for kidney stones compared with non-ADPKD patients.^78^ Kidney volume was significantly greater with nephrolithiasis than without; a kidney volume ≥500 ml was a significant predictor of nephrolithiasis in patients with ADPKD with normal renal function.^79^ The number of kidney cysts and the predominant cyst size are also significantly associated with nephrolithiasis in ADPKD.^80^

Because crystal deposition may accelerate cyst growth^81^ and hypocitraturia is associated with faster eGFR decline^82^ we have been evaluating for kidney stone risk factors by obtaining a 24-hour urine on patients with evidence of stones or mural calcification within a cyst (Table 3). Patients with ADPKD with stones were significantly more likely to be hypocitraturic with reduced 24-hour urine volume and urinary magnesium excretion, which are prolithogenic urinary risk factors.^80^ We treat with alkalinizing therapy as appropriate.

CT is the preferred imaging technique for stone evaluation.^79,83^ The presence of cysts within the sonographic field may degrade the diagnostic performance of ultrasound.^77^ CT is superior to ultrasound for exploration of kidney stones and detection of small stones, as well as detection of stones trapped in the ureters.^84^

Kidney stone management in patients with ADPKD generally follows the same guidance as management in patients without ADPKD. Ureteroscopy is safe and effective, with the advantage that it can be used in the presence of cyst-induced anatomic abnormalities and does not induce risk of traumatic nephron loss.^85^ Percutaneous surgical approaches are used in some cases with larger stones.^86^ Extracorporeal shock wave lithotripsy (ESWL) is the preferred and relatively least invasive intervention to directly treat stones, and it is best practice to use ESWL to treat stones smaller than 2 mm in adults with ADPKD. In addition, ESWL has also been shown to be an effective intervention in children with polycystic kidneys as one study reported a 94% stone-free status at a 12-month follow-up in 17 children with polycystic kidneys.^11^

Tolvaptan may confer protective effects against stone formation. A 2020 prospective, observational cohort study by Bargagli *et al.*^87^ found that urinary supersaturation ratios for calcium oxalate, brushite, and uric acid were significantly reduced with tolvaptan use. Higher rates of urinary citrate and calcium excretion, in conjunction with reduced net acid excretion, were observed in tolvaptan-treated patients with ADPKD.

## ICAs

ICAs may rupture leading to subarachnoid, intraventricular, or intracerebral hemorrhage.^88^ Ruptured ICAs account for 4%–7% of deaths in patients with ADPKD.^89^ In addition, there are case reports of unilateral and bilateral subdural hematomas in ADPKD.^90–93^ Some have been associated with arachnoid cysts.

The prevalence of ICAs is four times higher in patients with ADPKD than in the general population (8%–12% versus 2%–3%, respectively).^94^ The incidence of ICA in patients with ADPKD with a positive family history of hemorrhagic stroke or ICA is higher compared with patients with ADPKD lacking such a family history (11.6% versus 23.5%).^95^ Although there is consensus to screen patients with ADPKD with a family history, there is no consensus on whether to screen every patient with ADPKD.^94^

We most commonly use a noncontrast magnetic resonance angiogram for screening (Table 1), but other modalities may be preferred because of patient issues (metallic surgical clips, etc).

Screening for ICA with mitral regurgitation angiography in patients with ADPKD every 5 years with annual follow-up in detected cases is cost-effective regardless of the family history of ICA.^96^ The existing literature recommends initial screening by the age of 30 years—earlier, if there is a family history of ICA.^94^ After a negative study, repeat screening every 5 years was also cost effective,^96^ but there is no consensus in the literature about rescreening after a negative study, particularly in patients without a family history.

Presymptomatic screening is generally not recommended before age 18 years. Because the average age of progression to ESKD is 55 years, and some studies have postulated that little benefit might be obtained from screening individuals older than 50 years.^94^ However, because the risk of intracranial hemorrhage increases with kidney failure,^97^ we discuss screening for ICA as part of preparation for RRT.

Annual surveillance mitral regurgitation angiography is optimal in patients with incidentally detected ICAs.^96^ Because most detected aneurysms are small and at low risk of rupture, many patients with incidentally found aneurysms will need long-term follow-up imaging.^98^ We collaborate with neurosurgery and neurointerventional radiology for monitoring of these patients.

In conclusion, ADPKD is a systemic disorder affecting multiple organ systems. Because most patients will be followed by a nephrology team, for these complex patients, the nephrologist is often the primary gatekeeper, referring to other specialties as appropriate. Our practices are based in academic tertiary medical care systems with access to interventional radiology, pain management, hepatology, neurosurgery, genetics, urology, cardiology, liver surgery, and high-risk obstetric care. In addition, we are supported by dieticians, social workers, and genetic counselors comfortable with ADPKD care and work closely with our pediatric nephrology and transplant specialists. For patients with complex ADPKD, close integration of care is our goal. Extra-renal manifestations of ADPKD continue post–kidney transplant, and therefore, monitoring and management should be lifelong.
